# Indications for extrahepatic bile duct resection due to perineural invasion in patients with gallbladder cancer

**DOI:** 10.1186/s12957-019-1735-0

**Published:** 2019-11-30

**Authors:** Suguru Maruyama, Hiromichi Kawaida, Naohiro Hosomura, Hidetake Amemiya, Ryo Saito, Hiroki Shimizu, Shinji Furuya, Hidenori Akaike, Yoshihiko Kawaguchi, Makoto Sudo, Shingo Inoue, Hiroshi Kono, Daisuke Ichikawa

**Affiliations:** First Department of Surgery, Faculty of Medicine, University of Yamanashi, 1110 Shimokato, Chuo, Yamanashi, 409-3898 Japan

**Keywords:** Gallbladder cancer, Perineural invasion, Extrahepatic bile duct resection

## Abstract

**Background:**

The indications for extrahepatic bile duct (EHBD) resection remain a major controversy in the surgical management of patients with gallbladder cancer. On the other hand, perineural invasion (PNI) was reported as an important factor in patients with gallbladder cancer because gallbladder cancer cells frequently spread to the tissues surrounding the EHBD via perineural routes. We assessed the correlation of PNI with clinicopathological factors in patients with gallbladder cancer to elucidate EHBD resection indications specifically in patients with PNI.

**Methods:**

This retrospective study assessed the PNI status of 50 patients with gallbladder cancer who underwent curative resection and examined the correlation between the presence of PNI and clinicopathological factors.

**Results:**

Thirteen patients (26%) were PNI positive. PNI was significantly correlated with male sex, proximal-type tumor, lymphatic and vascular invasion, and advanced T stage. Multivariate analysis found that PNI positivity (*p* < 0.001), lymphatic invasion (*p* = 0.007), and nodal stage (*p* < 0.001) were independent prognostic factors. PNI was never observed in patients with stage T1 cancer. Conversely, PNI was detected rarely in distal-type tumors, all of whom developed various types of recurrences.

**Conclusions:**

These results clearly demonstrated the prognostic impact of PNI in patients with gallbladder cancer. We suggest that EHBD resection in combination with cholecystectomy may not be useful for distal-type tumors from a perspective of PNI.

## Introduction

Gallbladder cancer is recognized as one of the most aggressive tumors, with a dismal prognosis [1]; even after curative surgery, the prognosis ranges from 17 to 45% [2]. In addition to aggressive nature of cancer, the anatomic features of the gallbladder such as the absence of a submucosal layer and close proximity to the liver and the hepatoduodenal ligament can encourage progression and spread of the lethal disease [3, 4]. Curative surgical resection (R0) is the only treatment approach that can provide long-term survival, and procedures vary depending on the extent of the tumor spread [5]. Some studies recommend radical cholecystectomy with extrahepatic bile duct (EHBD) resection in patients with gallbladder cancer even in the absence of direct invasion to the hepatoduodenal ligament based on studies showing that gallbladder cancer cells frequently spread to the tissues surrounding the EHBD via perineural and lymphatic routes [6, 7]. In fact, the dense neural network comprising nerve fibers and plexuses circumvolutes EHBD. Furthermore, there is abundant nerve tissue surrounding the gallbladder and the bile duct [8]. Tumor cells can also spread through the perineural space. Importantly, perineural invasion (PNI) was reported as a significant prognostic factor in patients with gallbladder cancer [6, 9].

At our institution, as a principle, we have been performing radical cholecystectomy with EHBD resection in patients with gallbladder cancer except for those patients with mucosal cancer. In the current study, we assessed correlations between PNI and clinicopathological factors in patients with gallbladder cancer who underwent surgical resection with or without EHBD resection and elucidated the indications for EHBD resection with a focus on the clinical significance of PNI.

## Methods

### Patients

Between 2001 and 2017, 68 patients with gallbladder cancer underwent surgical resection at the University of Yamanashi Hospital. Patients who underwent non-curative resection were excluded from the study. Thus, 50 patients who underwent surgery were included in this retrospective study. We diagnosed all cases as gallbladder cancer using computed tomography (CT), magnetic resonance imaging (MRI), and endoscopic ultrasound (EUS) before surgery; therefore, there was no incidental cancer. None of the patients received preoperative chemotherapy or chemoradiotherapy. In principle, cholecystectomy with EHBD resection was performed in patients with gallbladder cancer except for those patients with mucosal cancer. The clinicopathological features of the cases were reviewed based on data recorded in the hospital database. Tumor specimens and resected lymph nodes were obtained at the time of surgery, fixed immediately in 10% neutral-buffered formalin, and embedded in paraffin. Macroscopic and microscopic classification of gallbladder cancer was based on the Union for International Cancer Control classification, 7th edition. Complications were defined using the Clavien classification, and grade ≥ 2 complications were recorded [10]. Tumors invading the neck or the cystic duct of the gallbladder were defined as proximal-type, and those localized in the body or the fundus were defined as distal-type. Circumferential tumor locations were categorized as hepatic and non-hepatic, and circumferential involvement was recorded as hepatic.

Postoperative follow-up comprised evaluation of hematological parameters, computed tomography, and ultrasonography. Follow-up procedures were performed every 3 months for at least 2 years and subsequently continued periodically for at least 5 years. The study was approved by the Ethics Committee of the Yamanashi University and performed in accordance with the ethical standards of the Declaration of Helsinki and its later amendments.

### Statistical analysis

Comparisons between two groups were made using the Student’s *t* test. Associations between PNI and categorical variables were evaluated using the *χ*^2^ test. Survival curves were constructed using the Kaplan–Meier method and compared using the log-rank test. Multivariate analyses of prognostic factors related to survival were performed using the Cox proportional hazards test. Statistical significance was set at a *p* < 0.05. All statistical analyses were performed with EZR (Saitama Medical Center, Jichi Medical University, Saitama, Japan), a graphical user interface for R (The R Foundation for Statistical Computing, Vienna, Austria) [11].

## Results

### The relationship of PNI with clinicopathological factors in patients with gallbladder cancer

**Fig. 1:**
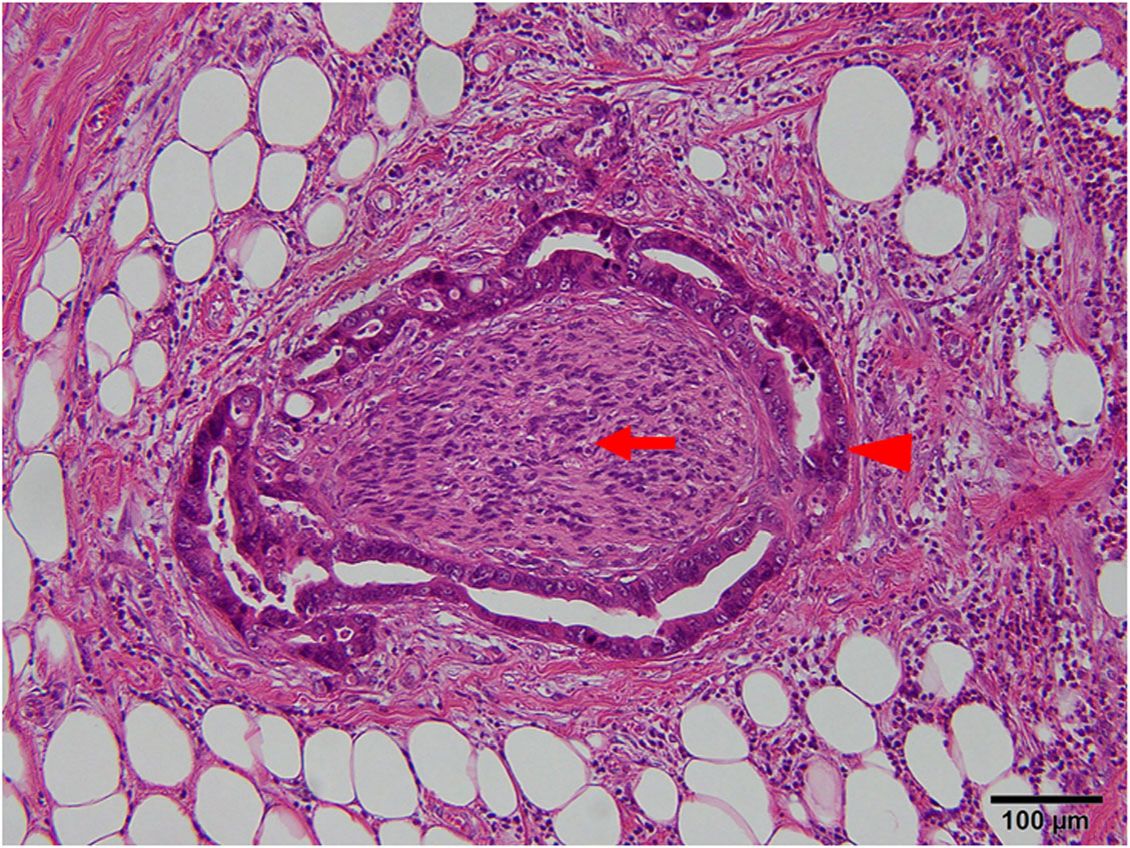
Representative photomicrographs of perineural invasion adjacent to tumor lesion; arrowhead indicates tumor and arrow indicates nerve

The clinicopathological characteristics of the patients included in the present study are summarized in Table 1. PNI adjacent to the tumor lesion was detected in 13 of the 50 cases (26.0%). Representative photomicrographic images of PNI are shown in Fig. 1. PNI was correlated significantly with male sex (*p* = 0.021), presence of postoperative chemotherapy (*p* < 0.001), presence of postoperative complications (*p* = 0.049), proximal-type tumor (*p* = 0.003), lymphatic invasion (*p* = 0.003), vascular invasion (*p* < 0.001), and advanced T stage (*p* = 0.010). PNI was not detected in patients with stage T1 cancer. PNI did not correlate with other clinicopathological factors such as N stage, numbers of resected lymph nodes, and lymph node ratio (Table 2).

**Table 1:** Clinicopathological characteristics of the 50 gallbladder cancer patients

Characteristics	Number of patients (%)
Age (years)	
< 70	23 (46.0)
70 ≦	27 (54.0)
Gender	
Male	17 (34.0)
Female	33 (66.0)
Size (mm)	
<35	26 (52.0)
35≦	24 (48.0)
Circumferential tumor location	
Hepatic side	32 (64.0)
No hepatic side	18 (36.0)
Tumor location	
Proximal	20 (40.0)
Distal	30 (60.0)
Surgical approach	
EHBD resection (-)	19 (38.0)
EHBD resection (+)	31 (62.0)
Postoperative complications	
No	46 (92.0)
Yes	4 (8.0)
Postoperative chemotherapy	
No	31 (62.0)
Yes	19 (38.0)
Liver resection	
No	20 (40.0)
Yes	30 (60.0)
T category	
T1	14 (28.0)
T2	29 (58.0)
T3	7 (14.0)
N category	
N (-)	37 (74.0)
N (+)	13 (26.0)
Perineural invasion	
No	37 (74.0)
Yes	13 (26.0)

*EHBD* extrahepatic bile duct

**Table 2:** Relationships between perineural invasion and clinicopathologic factors of gallbladder cancer patients

Characteristics	PNI (−)	PNI (+)	*p* value
	*n* = 37 (74.0%)	*n* = 13 (26.0%)	
Age (years)			
< 70	15 (40.5)	8 (61.5)	0.215
70 ≦	22 (59.5)	5 (38.5)	
Gender			
Male	9 (23.4)	8 (61.5)	0.021*
Female	28 (75.7)	5 (38.5)	
Postoperative chemotherapy			
No	29 (78.4)	2 (15.4)	< 0.001*
Yes	8 (21.6)	11 (84.6)	
Postoperative complications			
No	36 (97.3)	10 (76.9)	0.049*
Yes	1 (2.7)	3 (23.1)	
Size (mm)			
< 35	20 (54.1)	6 (46.2)	0.751
35 ≦	17 (45.9)	7 (53.8)	
Circumferential tumor location			
Hepatic side	21 (56.8)	11 (84.6)	0.098
No hepatic side	16 (43.2)	2 (15.4)	
Tumor location			
Proximal	10 (27.0)	10 (76.9)	0.003*
Distal	27 (73.0)	3 (23.1)	
Lymphatic invasion			
No	27 (73.0)	3 (23.1)	0.003*
Yes	10 (27.0)	10 (76.9)	
Vascular invasion			
No	32 (86.5)	3 (23.1)	< 0.001*
Yes	5 (13.5)	10 (76.9)	
T category			
T1	14 (37.8)	0 (0.0)	0.010*
T2, T3	23 (62.2)	13 (100.0)	
N category			
N (-)	29 (78.4)	8 (61.5)	0.281
N (+)	8 (21.6)	5 (38.5)	
Number of resected lymph nodes	6.32	5.00	0.494
Lymph node ratio	0.24	0.21	0.791

*PNI* perineural invasion. Significant differences between samples are indicated as *p <0.05

### The prognostic impact of HH

**Fig. 2:**
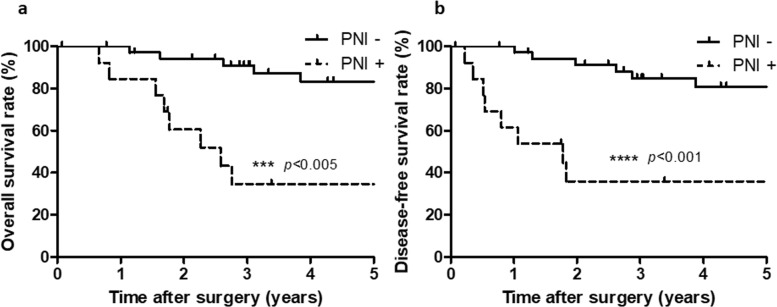
Kaplan–Meier curves for **a** overall survival rates and **b** disease-free survival rates. ***p < 0.005 compared with PNI (−) group using the log-rank test. ****p < 0.001 compared with PNI (−) group using the log-rank test. PNI perineural invasion

The median follow-up period of the study cohort was 63 months. Figure 2 shows the survival curves stratified according to the PNI status. Briefly, the overall and disease-free survival rates were significantly lower in the PNI-positive patients compared with the PNI-negative patients (*p* < 0.005 and *p* < 0.001, respectively).

Univariate analysis identified lymphatic and vascular invasion as well as the N stage as significant prognostic factors, whereas age, sex, tumor size, tumor location, EHBD resection, and the T stage were not found to be significantly associated with prognosis (Table 3). Subsequent multivariate analysis demonstrated that the presence of PNI was an independent prognostic factor (*p* < 0.001), as were lymphatic invasion (*p* = 0.007) and N stage (*p* < 0.001) (Table 3).

**Table 3:** Univariate and multivariate disease-free survival analyses of prognostic factors

	Univariate		Multivariate	
Characteristics	*p* value	Hazard ratio	95% CI	*p* value
Age years				
< 65	0.410			NA
65 ≦				
Gender				
Male	0.114			NA
Female				
Size (mm)				
< 35	0.433			NA
35 ≦				
Circumferential tumor location				
Hepatic side	0.073			NA
No hepatic side				
Tumor location				
Proximal	0.500			NA
Distal				
EHBD resection				
No	0.352			NA
Yes				
Liver resection				
No	0.706			NA
Yes				
Lymphatic invasion				
No	0.056			NA
Yes				
Vascular invasion				
No	0.018*	1		
Yes		1.465	0.201–3.015	0.717
T category				
T1	0.177			
T2, T3				NA
N category				
N (-)	< 0.001*	1		
N (+)		13.24	3.759–46.64	< 0.001*
Perineural invasion				
No	< 0.001*	1		
Yes		11.96	2.594–55.14	< 0.001*

*EHBD*, extrahepatic bile duct; *CI*, confidence interval; *NA*, not adopted. Significant differences between samples are indicated as *p <0.05

In this series, PNI was found in only three of the 30 cases with distal-type gallbladder cancer; all three patients were treated by cholecystectomy with EHBD resection. However, all patients developed various types of recurrences even after R0 resection; therefore, the extended procedures were found not to have clinical therapeutic efficacy in these patients.

## Discussion

Malignant tumors develop and progress via various routes of spread including hematogenous and lymphatic dissemination and local invasion. Local invasion is generally divided into direct invasion with destruction of the existing tissues and tumor spread through the loose space with particular histologic nature. As a representative of the latter, spreading through perineural space, i.e., PNI, is widely recognized as an important adverse pathological feature of many malignancies including pancreatic, prostate, and neck cancers [12, 13]. In these cancer types, the presence of PNI is a well-known poor prognostic factor [12, 13]. Similarly, PNI is detected frequently in gallbladder cancer and acknowledged for its clinical significance [6, 9].

PNI was detected more frequently in hepatic-sided and proximal-type gallbladder cancer in the current study cohort. Furthermore, PNI was an independent prognostic factor. However, our analysis indicated that PNI was not correlated with lymph node metastasis although lymphatic vessels and lymph nodes are adjacent to the nerves and plexuses around the gallbladder and the EHBD. Results from several recent experimental studies suggested that tumor cells might have increased affinity for nerve [14], implicating PNI in arising from a reciprocal interaction between the tumor cells and the microenvironment of the host nerve. The mechanisms of progression through nerve fibers and the lymphatic route might be distinct. To support this possibility, there was no significant correlation between PNI and specific recurrence patterns such as lymphatic or local recurrence in the current study cohort (data not shown).

Whether EHBD resection should be routinely performed in patients with gallbladder cancer remains controversial [5]. Some studies suggested that EHBD resection should be performed routinely during radical cholecystectomy [6, 15, 16], whereas others reported that EHBD resection did not improve prognosis [17–19]. D’ Angelica et al. suggested EHBD resection is appropriate when necessary to clear disease but are not mandatory in all cases [20]. We agree with this report. Moreover, they have reported that the median number of lymph nodes was similar regardless of whether EHBD resection had been performed, and lymphadenectomy plus EHBD resection was not associated with an improvement in survival [17, 20]. Therefore, routine EHBD resection was not associated with lymph node yield or survival. Recently, Kurahara et al*.* found that EHBD resection improved prognosis in patients with proximal-type gallbladder cancer [21].

Considering that EHBD resection in combination with cholecystectomy is recognized as extended wide resection for cases with spread through PNI, EHBD resection is not necessary for patients with stage T1 gallbladder cancer with no evidence of PNI. Conversely, PNI was rarely detected in distal-type gallbladder cancers. The lower frequency of PNI in distal-type tumors might be due to the sparse nerve networks in the distal lesion or might reflect a biological feature. Intraoperative pathological diagnosis for the proximal margin should be useful for the decision for performing EHBD resection. However, PNI cannot be diagnosed before surgery. Therefore, it is important to know that PNI is detected more frequently in proximal-type gallbladder cancer and rarely detected in distal-type cancer.

All patients with PNI-positive distal-type gallbladder cancer developed recurrence despite the EHBD resection. In this study, there were no cases of R1 resection for not performing EHBD; however, two cases with PNI-positive distal-type gallbladder cancer were R1 resection despite performing EHBD resection (data not shown). These results indicate that EHBD resection in combination with cholecystectomy failed to provide prognostic benefit for those with distal-type gallbladder cancer. However, the number of distal-type gallbladder cancer was small in this study, and further studies are warranted to confirm that. If patients with PNI-positive distal-type gallbladder cancer obtain long-term prognosis by performing EHBD resection, EHBD resection should be performed even for patients with distal-type cancer.

To the best of our knowledge, no studies to date evaluated the clinical significance of EHBD resection for gallbladder cancer in the context of PNI. Magnon et al. demonstrated that surgical sympathectomy prevented the early-phase prostate cancer development [22], whereas Zhao et al. demonstrated that surgical denervation of the stomach markedly reduced gastric tumor incidence and progression [23]. Total resection of the nerve tissues around the EHBD should be discussed in two aspects: survival benefit with total removal of the tumor cells around the nerve tissues and potential post-denervation effects on tumor development and progression. The current study demonstrated that EHBD resection in combination with cholecystectomy may not provide any overt survival benefits at least for certain subsets of patients with gallbladder cancer. However, this study has certain limitations. The number of cases analyzed was small, and multi-center large-scale investigations are necessary to confirm these results.

## Conclusions

In conclusion, the current study clearly demonstrated the prognostic impact of PNI in patients with gallbladder cancer. We suggest that EHBD resection in combination with cholecystectomy may not be useful at least in patients with stage T1 disease and distal-type tumors from a perspective of PNI.

## Data Availability

Data were collected at University of Yamanashi and are not publicly available.

## Abbreviations

EHBD: Extrahepatic bile duct
PNI: Perineural invasion
